# Nitric oxide-dependent long-term depression but not endocannabinoid-mediated long-term potentiation is crucial for visual recognition memory

**DOI:** 10.1113/jphysiol.2013.254862

**Published:** 2013-05-13

**Authors:** Francesco Tamagnini, Gareth Barker, E Clea Warburton, Costanza Burattini, Giorgio Aicardi, Zafar I Bashir

**Affiliations:** 1 School of Physiology and Pharmacology, Medical Research Council Centre for Synaptic Plasticity, Bristol University Bristol, UK; 2 Dipartimento di Fisiologia Umana e Generale, Università di Bologna Bologna, Italia; 3 Centro Interdipartimentale ‘Luigi Galvani’ per lo studio integrato della Biofisica, della Bioinformatica e della Biocomplessità Bologna, Italia

## Abstract

Synaptic plasticity in perirhinal cortex is essential for recognition memory. Nitric oxide and endocannabinoids (eCBs), which are produced in the postsynaptic cell and act on the presynaptic terminal, are implicated in mechanisms of long-term potentiation (LTP) and long-term depression (LTD) in other brain regions. In this study, we examine these two retrograde signalling cascades in perirhinal cortex synaptic plasticity and in visual recognition memory in the rat. We show that inhibition of NO-dependent signalling prevented both carbachol- and activity (5 Hz)-dependent LTD but not activity (100 Hz theta burst)-dependent LTP in the rat perirhinal cortex *in vitro*. In contrast, inhibition of the eCB-dependent signalling prevented LTP but not the two forms of LTD *in vitro*. Local administration into perirhinal cortex of the nitric oxide synthase inhibitor NPA (2 μm) disrupted acquisition of long-term visual recognition memory. In contrast, AM251 (10 μm), a cannabinoid receptor 1 antagonist, did not impair visual recognition memory. The results of this study demonstrate dissociation between putative retrograde signalling mechanisms in LTD and LTP in perirhinal cortex. Thus, LTP relies on cannabinoid but not NO signalling, whilst LTD relies on NO- but not eCB-dependent signalling. Critically, these results also establish, for the first time, that NO- but not eCB-dependent signalling is important in perirhinal cortex-dependent visual recognition memory.

**Key points**

- Perirhinal cortex (Prh) is critically involved in visual recognition memory and synaptic plasticity.
- Nitric oxide and endocannabinoids (eCBs) have been shown to act as retrograde messengers in synaptic plasticity in several brain areas, but no study has yet investigated their role in synaptic plasticity in Prh.
- Evidence is still lacking of a retrograde messenger involved in synaptic plasticity in Prh.
- In this study, we show that NO is involved in long-term depression (LTD) but not in long-term potentiation (LTP). Conversely, eCBs are involved in LTP but not in LTD. Crucially, inhibiition of NO signalling prevents visual recognition memory acquisition, whilst inhibition of eCB signalling does not affect recognition memory.
- These results suggest that LTD but not LTP is a neuronal correlate of visual recognition memory.

## Introduction

The perirhinal cortex (Prh) is essential for the ability to discriminate between novel and familiar individual stimuli (Brown & Aggleton, 2001), and the processes underlying activity-dependent synaptic plasticity in Prh may provide clues about the cellular and molecular correlates of this component (i.e. familiarity discrimination) of recognition memory (Warburton *et al.* 2003, 2005; Griffiths *et al.* 2008; Massey *et al.* 2008; Seoane *et al.* 2009; Brown *et al.* 2010). Retrograde signalling is critical in synaptic plasticity, co-ordinating pre- and postsynaptic changes following induction of long-term potentiation (LTP) or long-term depression (LTD). Whilst roles for NO and endocannabinoids (eCBs) as retrograde messengers in synaptic plasticity have been demonstrated previously, there is no known role of NO or eCBs in Prh synaptic plasticity.

In physiological conditions, NO is synthesized postsynaptically in neurones and blood vessels by constitutive isoforms of nitric oxide synthase (neuronal, nNOS; endothelial, eNOS) that are activated by Ca^2+^–calmodulin (reviewed by Garthwaite & Boulton, 1995; Garthwaite, 2008; Steinert *et al.* 2010). Nitric oxide can play a role in retrograde signalling in LTD in the cerebellum, hippocampus and prefrontal cortex (Reyes-Harde *et al.* 1999; Shin & Linden, 2005; Huang & Hsu, 2010) and in LTP in the hippocampus and visual cortex (Arancio *et al.* 1995, 1996, 2001; Wang *et al.* 2005; Haghikia *et al.* 2007). Furthermore, NO has been implicated in learning and memory, including spatial (Böhme *et al.* 1993) and motor learning (Allen & Steinmetz 1996; Nagao *et al.* 1997).

Endocannabinoids are normally synthesized following postsynaptic stimulation of G_q_-coupled receptors by a variety of different neurotransmitters. In the CNS, eCBs decrease transmitter release through activation of presynaptic cannabinoid receptor 1 (CB1). Furthermore, eCBs have been implicated in mechanisms of LTD in the striatum, cortex and hippocampus (Robbe *et al.* 2002; Lafourcade *et al.* 2007; Sergeeva *et al.* 2007; Yasuda *et al.* 2008) and in hippocampal and amygdala-dependent associative learning and memory (Marsicano *et al.* 2002; Varvel *et al.* 2007).

Interestingly, there is no evidence concerning the role of retrograde signalling systems in Prh synaptic plasticity and so the link between these signalling systems and Prh-dependent learning is still to be established. Therefore, in this study we address the roles of NO- and eCB-dependent signalling in both LTP and LTD in Prh *in vitro* and in visual recognition memory *in vivo*. We demonstrate that inhibition of nitric oxide synthase (NOS) and of soluble guanylate cyclase (sGC) prevents LTD but not LTP and that inhibition of cannabinoid signalling, by bath application of AM251 (1 μm), a CB1 antagonist, prevents LTP but not LTD *in vitro*. We then show that inhibition of NOS but not inhibition of CB1 receptors impairs the familiarity discrimination component of recognition memory. These data suggest a reciprocal involvement of NO and eCBs in perirhinal LTD and LTP, respectively, and point to a role for NO in visual recognition memory acquisition, giving further confirmation that depression-like phenomena in Prh may represent the cellular correlate of this form of memory, as previously suggested (Warburton *et al.* 2003; Griffiths *et al.* 2008; Massey *et al.* 2008; Seoane *et al.* 2009).

## Methods

### Animals

Adult male pigmented (Dark Agouti, DA) rats (220–250 g; Bantin and Kingman, Hull, UK), for *in vivo* experiments, and postnatal day 28–35 male DA (Bantin and Kingman, Hull, UK) or albino rats (Sprague–Dawley, SD; Charles River, Margate, UK), for *in vitro* electrophysiology, were maintained on a 12 h light–12 h dark cycle, with the dark phase during normal daylight. All experiments were performed in accordance with the UK Animals (Scientific Procedures) Act 1986 and the European Community Guidelines on animal care, and had the approval of the Ethical Review Committees of the Universities of Bristol and Bologna.

### *In vitro* experiments

#### Slice preparation

Each animal was anaesthetized with a mixture of oxygen and isoflurane or halothane and subsequently decapitated. The brain was rapidly removed and placed in ice-cold (2–4°C), oxygenated (95% O_2_–5% CO_2_) artificial cerebrospinal fluid (aCSF) containing (mm): 125 NaCl, 2.5 KCl, 1.2 NaH_2_PO_4_, 1.2 MgCl_2_, 2.4 CaCl_2_, 26 NaHCO_3_ and 11 glucose. The cerebellum and the frontal and parietal lobes were removed with single scalpel cuts. The sample was then glued on a stainless-steel stage and immediately placed in the slicing chamber of a vibratome (WPI Europe, Berlin, Germany) filled with ice-cold, oxygenated aCSF. Horizontal slices (400 μm thick), comprising hippocampus, Prh and lateral entorhinal cortex, were obtained and then left to recover (60–90 min) in oxygenated aCSF at room temperature. After recovery, one single slice was placed in a submerged recording chamber, maintained at 32°C and continuously perfused with oxygenated aCSF delivered at a flow rate of 2–3 ml min^−1^.

#### Electrophysiological recordings

After acclimatization (at least 30 min), square current pulses (duration 0.2 ms) were applied every 30 s (0.033 Hz) via a stimulating electrode placed in the Prh superficial layers (approximately layer II/III); the stimulus intensity was chosen in order to induce 50–60% of the maximal synaptic response. The subsequently evoked field excitatory postsynaptic potentials (fEPSPs) were recorded in the same layers with a glass micropipette (3–5 MΩ) recording electrode, containing 2 m NaCl solution, connected through a silver chloride wire to an amplifier (Axopatch 200, Axon Instruments, Foster City, CA, USA; or EPC-7, HEKA, Lambrecht, Germany). Single sweeps (100 ms) were digitally acquired with an analog/digital (A/D) board (National Instruments or Digidata 1200, Axon Instruments, PA, USA), transferred to a PC and visualized via the acquisition and analysis software WinLTP (Anderson and Collingridge, 2007) or Axoscope (Axon instruments, PA, USA). After the acquisition of a stable baseline (at least 10–30 min) in control conditions or after drug pre-application, one of the following stimulation protocols was applied: (i) 100 Hz theta-burst stimulation (100 Hz-TBS) to induce LTP (see Aicardi *et al.* 2004); (ii) low-frequency stimulation (3000 pulses delivered at 5 Hz; 5 Hz-LFS) to induce activity-dependent LTD; (iii) weak 5 Hz-LFS (1350 pulses delivered at 5 Hz) to induce an activity-dependent transient depression; or (iv) bath application of carbachol (CCh; 50 μm, 10 min) to induce LTD (Massey *et al.* 2001). Evoked fEPSPs in layer II/III of Prh may show a more complex shape compared with other brain areas (i.e. hippocampal Schaffer collateral to CA1 synapses), due to the contamination of synaptic and non-synaptic components from different cortical layers. At the end of all experiments, solution containing zero added calcium was applied to remove all synaptic responses. In these conditions, only non-synaptic responses remained. Therefore, the experiment was subsequently re-analysed to measure only the synaptic field response; typically, the latency of the peak synaptic component was >4 ms from the end of the stimulus artefact, although this varied between experiments. Each sweep was analysed online and offline with the software WinLTP and normalized for the baseline value, calculated as the mean of the fEPSP amplitudes recorded in the baseline period corresponding to the first 10–30 min of the experiment, prior to the application of drugs and/or stimulation protocols. All the experimental groups were plotted as mean values ± SEM. The effects of the conditioning protocols were measured 50–60 min after induction of LTP or LTD, corresponding to the last time period of the experiment, unless otherwise stated. Significance from baseline was calculated between the last time point of the baseline and the last point of follow-up (50–60 min) and evaluated using Student's paired *t* test or one way repeated measures ANOVA, as appropriate; Student's unpaired *t* tests or one-way ANOVA were used, as appropriate, for comparisons between experimental groups. The number of experiments indicated for each experimental group is relative to the number of animals used (i.e. *n*= 8 means 8 slices from 8 animals).

**Figure 4:**
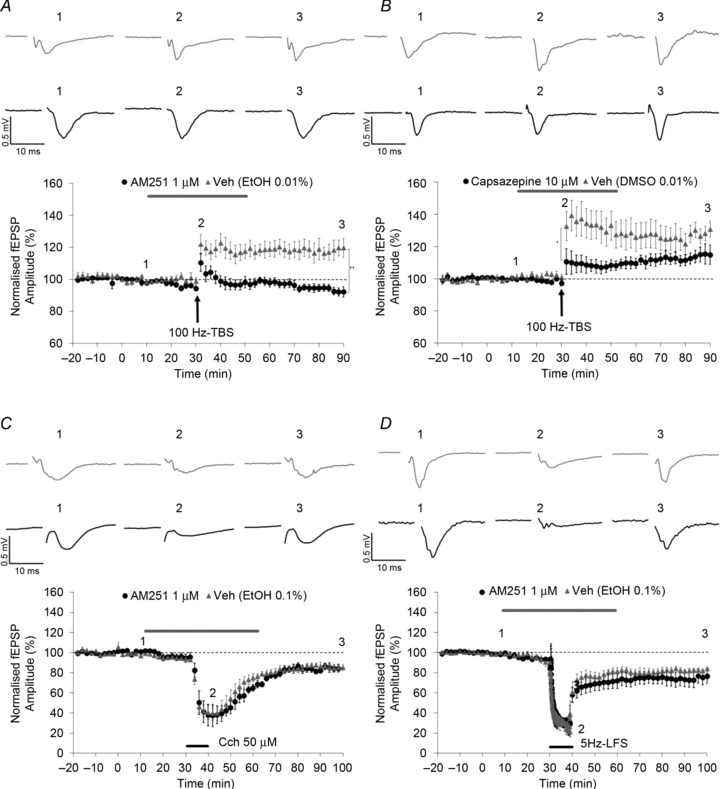
Endocannabinoid involvement in induction of perirhinal cortex (Prh) LTP but not LTD The pre-application of the CB1 antagonist AM251 (1 μm, *A*; *n*= 8, Student's paired *t* test, *P* > 0.05) blocked 100 Hz-TBS-LTP induction. The TRPV1 antagonist capsazepine (10 μm) blocked the first phase of LTP induction (one-way repeated measures ANOVA, 10 μm, *B*; *n*= 6, *P* < 0.01). AM251 (1 μm) did not affect CCh-LTD induction (*C*; *n*= 7, Student's paired *t* test, *P* < 0.01) and 5 Hz-LTD induction (*D*; *n*= 5, Student's paired *t* test, *P* < 0.01).

Control experiments for 5 Hz-LFS LTD, CCh LTD, 100 Hz-TBS LTP and weak 5 Hz-LFS + diethylamine-NONOate (DEA/NO) LTD were interleaved to each treatment on separate slices and performed in the presence of 0.1%DMSO or 0.1% EtOH or pure aCSF, depending on the solvent used to prepare the drug stock solution. Given that no significant differences were observed amongst the different solvents, all controls were plotted together for each stimulation protocol. For the purposes of clarity, in Fig. 4 each experiment is shown with its interleaved vehicle control.

#### Drugs

The non-selective NOS inhibitor l-*N*^G^-nitroarginine methyl ester hydrochloride (l-NAME) was purchased from Sigma-Aldrich S.r.L, Milan, Italy and maintained at −20°C. The NOS selective antagonist *N*^G^-propyl-l-arginine (NPA) was purchased from Tocris Cookson (Bristol, UK), dissolved in 0.9% saline solution at a stock concentration of 20 mm and kept refrigerated at −20°C.

The sGC inhibitor 4*H*-8-bromo-1,2,4-oxadiazolo [3,4-d]benz[b][1,4]oxazin-1-one (NS2028) was purchased from Sigma-Aldrich (S.r.L., Italy), dissolved in DMSO in a 20 mm stock solution and maintained at −20°C.

The NO donor 2-(*N*,*N*-diethylamino)-diazenolate 2-oxide sodium salt hydrate (DEA/NO) was purchased from Sigma-Aldrich (S.r.L., Italy) and maintained at −20°C. Stock solutions (3 mm) were prepared daily by dissolving DEA/NO in NaOH 10 mm. Artificial cerebrospinal fluid containing DEA/NO (3 μm) was prepared immediately before the bath application by 1:1000 dilution of stock solution in aCSF (half-life of DEA/NO is 16 min at pH 7.4 and 21°C and 6 min at pH 7.4 and 32°C).

The cholinergic agonist 2-hydroxyethyltrimethyl ammonium chloride carbamate (carbachol) was purchased from Sigma-Aldrich (S.r.L., Italy) and maintained at room temperature. Stock solutions (50 mm) in H_2_O were stored at −20°C.

The CB1 receptor selective antagonist *N*- (piperidin-1-yl)-5-(4-iodophenyl)-1-(2,4-dichlorophenyl) -4-methyl-1*H*-pyrazole-3-carboxamide (AM251) was purchased from Tocris Cookson (Bristol, UK), dissolved in pure ethanol 1 mm stock solutions and maintained at −20°C.

The Transient receptor potential cation channel subfamily V member 1 (TrpV1) receptor antagonist capsazepine was purchased from Tocris Cookson (Bristol, UK), dissolved in DMSO in a 10 mm stock solution and maintained at −20°C.

Fresh solutions of each drug at their final concentrations were prepared daily in aCSF for electrophysiology and in 0.9% saline for *in vivo* experiments.

### *In vivo* experiments

#### Surgical implantation of cannulae into perirhinal cortex

Cannula implantation was carried out in rats (*n*= 10) deeply anaesthetized with isoflurane (Merial Animal Health Ltd., Harlow, UK) and placed in a stereotaxic frame, where the skull was maintained in a flat position (the height difference between bregma and lamda was <0.5 mm). Two stainless-steel guide cannulae (26 gauge, Plastics One Inc., Roanoke, VA, USA, via Semat in UK) were implanted through holes in the skull, at an angle of 20 deg to the vertical and according to the following co-ordinates (relative to bregma): anteroposterior −5.6 mm, lateral ±4.5 mm and ventral −6.7 mm (relative to the skull surface; Paxinos & Watson, 1986). The guide cannulae were anchored to the skull with two stainless-steel screws and dental cement (CMW1 Radiopaque with gentamicin, DePuy International Ltd, Blackpool, UK). Cannulae were kept patent with obdurators (Plastics One Inc.) except at the time of the infusion. The rats were allowed to recover for at least 14 days before the experiment began.

#### Infusions

The drugs used were the nNOS inhibitor NPA or the CB1 receptor inhibitor AM251 dissolved as described above (see ‘*Drugs*’). Vehicle infusions were either saline as the control for NPA or saline containing 0.1% EtOH as the control for AM251. The NPA was infused at a dose of 2 μm; the AM251 was infused at a dose of 10 μm. Bilateral infusions were made into the Prh through a 33 gauge cannula (Plastics One Inc.), which protruded 1 mm beyond the tip of the guide cannula. Each infusion cannula was attached to a Hamilton syringe (Hamilton Bonaduz, Bonaduz, Switzerland) via PVC tubing (Barloworld Scientific Ltd, Maidenhead, UK). The syringe was advanced with an infusion pump (Harvard Bioscience, Holliston, MA, USA) to produce an infusion rate of 0.5 μl min^−1^ for 2 min, and 5 min later the injection cannulae were withdrawn.

#### Behavioural testing: novel object preference task

The methodology of the novel object preference test has been described in detail in previous studies (Warburton *et al.* 2003; Barker *et al.* 2006*a*,*b*, 2007). In brief, this task took place in an arena (50 cm × 90 cm × 100 cm). The walls around the arena were painted black and were surrounded with black curtains and with sawdust on the floor. The rat's behaviour was monitored using a camera and a video recorder. The objects were made of Duplo bricks (LegoProduktion A.G., Baar, Switzerland) and varied in size (ranging from 8 cm × 7 cm × 5 cm to 25 cm × 15 cm × 10 cm), colour and shape, and were placed near the two corners at either end of one side of the arena (15 cm from each adjacent wall). Prior to the start of memory testing, each rat was habituated to the empty arena for 5 min daily for 4 days. The novel object preference test comprised two phases, acquisition and test, separated by a delay of 20 min or 24 h. In the acquisition phase, each animal was allowed to explore two identical objects for 40 s of exploration or a maximum of 4 min spent in the arena. Following the period of exploration, the rat was removed from the arena and placed in a holding cage for the duration of the retention delay. In the test phase, the rat was replaced in the arena and allowed to explore an identical third copy of the object explored in the acquisition phase and a novel object for a total of 3 min. In both acquisition and test phases, the time spent exploring each of the objects was recorded. Exploration was considered only when the animal's nose was directed towards the object at a distance of less than 1 cm. If the time of exploration was <15 s in the acquisition phase or <10 s in the test phase, the animal was discarded from the analysis of that experiment. In order to avoid biases linking the objects and their position in the arena, these two parameters were counterbalanced between animals in a group and between control and drug-treated animals. The experimenter was blinded concerning the treatment of each animal.

##### Experimental design

Animals were administered drug or vehicle locally into the Prh, starting 15 min before the commencement of the acquisition phase, and after a minimum of 48 h, drug or vehicle was infused in a cross-over design and the animal was tested again. To evaluate shorter-term and long-term memory, delays between acquisition and test phases of 20 min and 24 h were used.

### Data analysis

All measures of exploration were made with the experimenter blinded to the drug status of each animal. Discrimination between the objects was determined using a discrimination ratio, calculated as the difference in time spent by each animal exploring the novel compared with the familiar object divided by the total time spent exploring both objects. This measure therefore takes into account individual differences in the total amount of exploration between rats (Ennaceur & Delacour, 1988; Dix & Aggleton, 1999). Comparisons were made using a multifactor ANOVA followed by *post hoc* pairwise comparisons. Additional analyses in both experiments examined whether individual groups had discriminated between the objects, using a one-sample *t* test comparing the discrimination ratio against chance performance.

### Histology

At the end of the experiment, rats were anaesthetized with pentabarbital (Euthatal, Rhône Mérieux, Toulouse, France) and perfused transcardially with 4% paraformaldehyde in 0.1 m phosphate buffer (pH 7.4). The brain was postfixed in paraformaldehyde for at least 24 h before being transferred to 30% sucrose in 0.1 m phosphate buffer for at least 48 h. Coronal sections were cut at 40 μm on a cryostat and stained with Cresyl Violet to examine cannula locations.

## Results

### Role of nitric oxide signalling in carbachol-dependent LTD in perirhinal cortex

**Figure 1:**
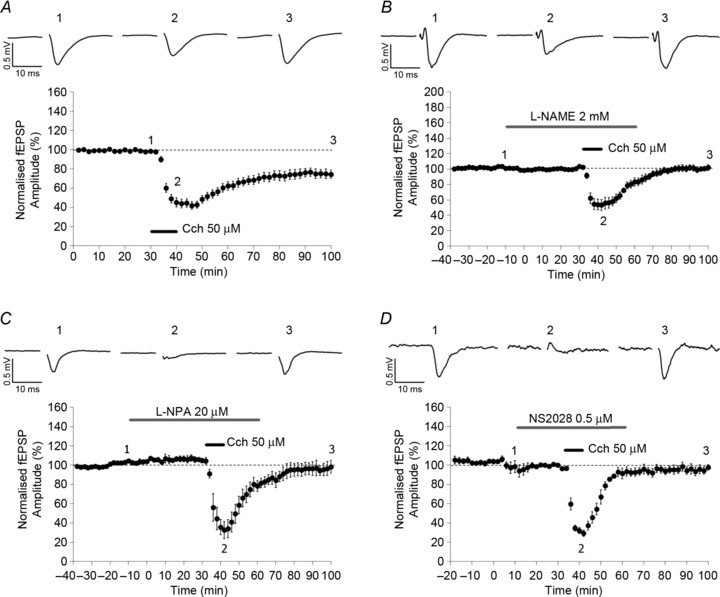
Involvement of nitric oxide synthase (NOS) and soluble guanylate cyclase (sGC) in carbachol (CCh) induction of long-term depression (LTD) The application of CCh (50 μm) resulted in the induction of a robust and prolonged LTD (*A*; *n*= 23, Student's paired *t* test, *P* < 0.01). Pre-application of the NOS non-selective antagonist l-NAME at 2 mm (*B*; *n*= 5, Student's paired *t* test, *P* > 0.05) blocked CCh-LTD induction. Pre-application of the selective antagonist for the neuronal isoform of NOS (nNOS) NPA (20 μm) blocked CCh-LTD induction (*C*; *n*= 5, Student's paired *t* test, *P* > 0.05). Pre-application of the sGC antagonist NS2028 (0.5 μm) blocked CCh-LTD induction (*D*; *n*= 6, Student's paired *t* test, *P* > 0.05). Data are plotted as mean normalized amplitudes ± SEM.

Extracellular fEPSPs were recorded in the superficial layers (approximately layer II/III) of Prh as previously described (Bilkey, 1996; Ziakopoulos *et al.* 1999; Aicardi *et al.* 2004). Consistent with previous observations (Massey *et al.* 2001), the bath application of carbachol (10 min; 50 μm) resulted in the induction of a large acute depression (Fig. 1*A*; depression to 45.4 ± 4.7% of baseline, tested at the last time point of CCh application, one-way repeated measures ANOVA, *P* < 0.01), followed by robust and prolonged LTD (CCh-LTD; Fig. 1*A*; *n*= 23, depression to 74.5 ± 4.4% of baseline, one-way repeated measures ANOVA, *P* < 0.01).

To investigate the role of NO-dependent signalling in CCh-LTD, the NOS inhibitor l-NAME was bath applied at different concentrations (at least 40 min before CCh application). In the presence of 200 μm l-NAME, CCh-LTD was blocked (data not shown; *n*= 9, 99.4 ± 4.1%, one-way repeated measures ANOVA, *P* > 0.05). In addition, 2 mm l-NAME also blocked CCh-LTD (Fig. 1*B*; *n*= 5, 101.9 ± 3.8%, one-way repeated measures ANOVA, *P* > 0.05). To further confirm the role of nitric oxide in CCh-LTD, we used an alternative NOS inhibitor, NPA. This compound has been variously reported to be a selective antagonist of (Zhang *et al.* 1997) or to show little selectivity (Pigott *et al.* 2013) for nNOS. Pre-application of NPA (20 μm) also blocked the induction of CCh-LTD (Fig. 1*C*; *n*= 5, 98.2 ± 6.7%, one-way repeated measures ANOVA, *P* > 0.05). Nitric oxide is known to activate cGMP synthesis by activation of sGC. Therefore, we applied the inhibitor of sGC, NS2028 (0.5 μm), and this blocked CCh-LTD (Fig. 1*D*; *n*= 6, 97.7 ± 2.9%, one-way repeated measures ANOVA, *P* > 0.05). The pre-application of each drug did not significantly affect the magnitude of depression in the acute phase of CCh application (see Table 1). For all the drugs tested, the blockade of CCh-LTD was significant compared with controls (one-way ANOVA, *F*= 6.505, *P* < 0.01; Holm–Sidak *post hoc* comparisons of each group *vs.* control group, *P* < 0.05). The application of each drug (except carbachol) did not affect basal synaptic transmission.

**Table 1:** Acute depression induced by bath application of carbachol (50 μm)

Treatment	Acute effects (mean % field EPSP ± SEM)	Significance *vs.* baseline (Student's paired *t* test)	Significance *vs.*controls (Student's unpaired *t* test)
Controls, *n*= 21	45.4 ± 4.7	*P* < 0.01	—
l-NAME (200 μm), *n*= 9	44.9 ± 4.2	*P* < 0.01	*P* > 0.05
l-NAME (2 mm), *n*= 5	53.8 ± 6.9	*P* < 0.01	*P* > 0.05
NPA (20 μm), *n*= 5	33.0 ± 6.4	*P* < 0.01	*P* > 0.05
NS2028 (0.5 μm), *n*= 6	32.1 ± 7.5	*P* < 0.01	*P* > 0.05
AM251 (1 μm), *n*= 7	28.4 ± 3.9	*P* < 0.01	*P* > 0.05

This table summarizes the acute effects of the bath application of carbachol (50 μm) on perirhinal cortex synaptic transmission in control conditions or after the pre-application of drugs. Each treatment did not affect the magnitude of the acute depression induced by carbachol compared with controls.

### Role of nitric oxide signalling in activity-dependent LTD in perirhinal cortex

**Figure 2:**
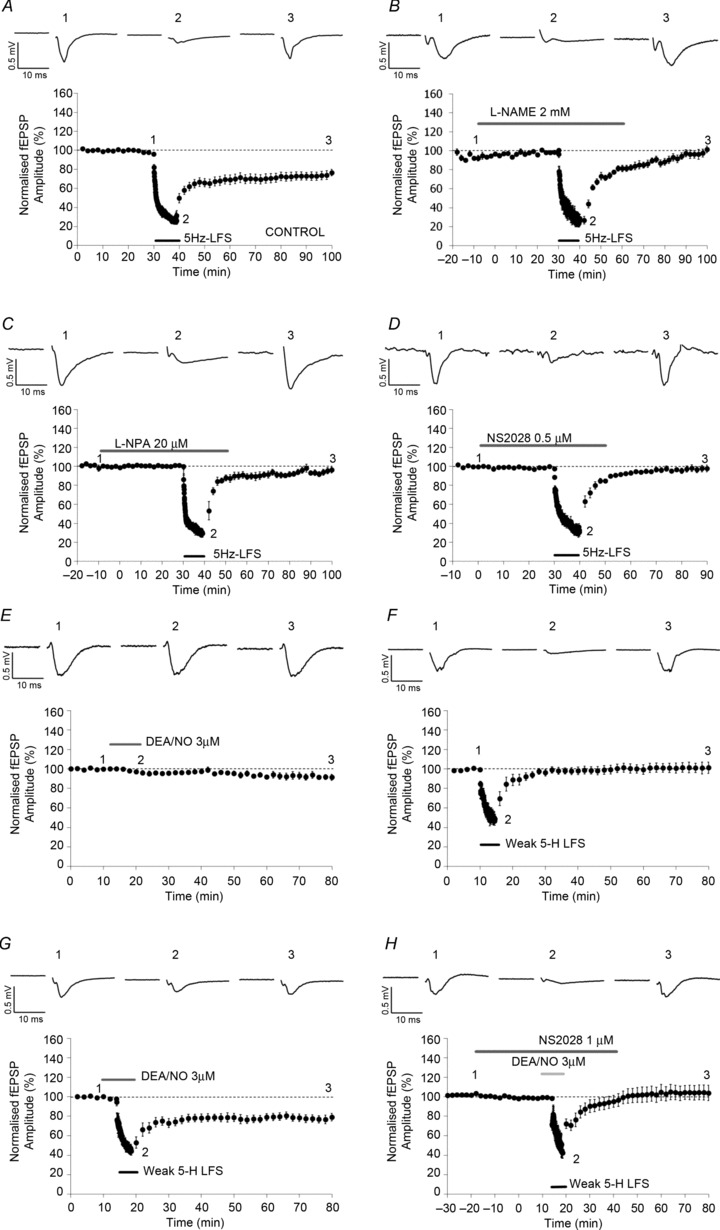
Involvement of NOS and sGC in 5 Hz-LTD induction The application of a low-frequency stimulation (LFS) consisting of 3000 pulses delivered at 5 Hz (5 Hz-LFS) resulted in the induction of a robust and prolonged LTD (*A*; *n*= 19, Student's paired *t* test, *P* < 0.01). Pre-application of the NOS non-selective inhibitor l-NAME (2 mm) blocked the induction of 5 Hz-LTD (*B*; *n*= 7, Student's paired *t* test, *P* > 0.05). Pre-application of the nNOS selective inhibitor NPA (20 μm) blocked the induction of 5 Hz-LTD (*C*; *n*= 6, Student's paired *t* test, *P* > 0.05). The 5 Hz-LTD induction was also blocked when the sGC antagonist NS2028 (0.5 μm) was pre-applied (*D*; *n*= 7, Student's paired *t* test, *P* > 0.05). The application of the NO donor DEA/NO (3 μm) for 10 min did not affect basal synaptic transmission (*E*; *n*= 5, Student's paired *t* test, *P* > 0.05), and the application of subthreshold 5 Hz-LFS (consisting of 1350 pulses instead of 3000; weak 5 Hz-LFS) induced a transient but not long-term depression of synaptic transmission (*F*; *n*= 12, Student's paired *t* test, *P* > 0.05). The co-application of the NO donor DEA/NO for 10 min and the weak 5 Hz-LFS, started after 5 min of bath application of DEA/NO, resulted in the induction of a robust and prolonged LTD (*G*; *n*= 13, Student's paired *t* test, *P* < 0.01). Pre-application of the sGC antagonist NS2028 (1 μm) blocked the induction of LTD by the co-application of DEA/NO and the weak 5 Hz-LFS (*H*; *n*= 9, Student's paired *t* test, *P* > 0.05).

The application of low-frequency stimulation consisting of 3000 pulses delivered for 10 min at 5 Hz (5 Hz-LFS) resulted in the induction of robust and prolonged LTD (5 Hz-LTD; Fig. 2*A*; *n*= 19, 76.6 ± 3.5%, Student's paired *t* test, *P* < 0.01), as previously reported (Aicardi *et al.* 2004; Massey *et al.* 2004; Jo *et al.* 2006). This form of LTD has previously been shown to depend on muscarinic M_1_ receptors in juvenile rats (Jo *et al.* 2006). Therefore, given the block of CCh-LTD by NOS inhibitors (see Fig. 1), in the next series of experiments we examined whether there is a role for NO or sGC in this activity-dependent form of LTD. Application of either either l-NAME (2 mm) or NPA (20 μm) prevented induction of 5 Hz-LTD (Fig. 2*B*; l-NAME 2 mm, *n*= 7, 98.9 ± 3.0%, Student's paired *t* test, *P* > 0.05; and Fig. 2*C*; NPA 20 μm, *n*= 6, 96.2 ± 3.1%, Student's paired *t* test, *P* > 0.05). In addition, the pre-application of the sGC inhibitor NS2028 (0.5 μm) also blocked 5 Hz-LTD induction (Fig. 2*D*; NS2028 0.5 μm, *n*= 7, 97.9 ± 3.5%, Student's paired *t* test, *P* > 0.05). For all the drugs tested, the blockade of 5 Hz-LTD was significant compared with controls (one-way ANOVA, *F*= 5.559, *P* < 0.03; Holm–Sidak *post hoc* comparisons of each drug group *vs.* control group, *P* < 0.05).

The potential role of NO-dependent signalling in 5 Hz-LTD was further confirmed in a second series of experiments. Bath application of the NO donor DEA/NO for 10 min did not affect basal synaptic transmission (Fig. 2*E*; *n*= 5, 94.3 ± 1.0%, Student's paired *t* test, *P* > 0.05). Note that NO release from DEA/NO follows pH- and temperature-dependent kinetics; in the experimental conditions chosen for this study (32°C and pH 7.4) the half-life of DEA/NO (6 min) is consistent with the application time of 10 min (Bon & Garthwaite, 2001). The application of a weak 5 Hz-LFS, consisting of 1350 pulses delivered at 5 Hz (weak 5 Hz-LFS) resulted in transient depression (Fig. 2*F*; *n*= 12, 101.3 ± 5.9%, Student's paired *t* test, *P* > 0.05). However, co-application of DEA/NO (3 μm) and weak 5 Hz-LFS resulted in a robust and prolonged LTD (Fig. 2*G*; *n*= 13, 79.1 ± 3.3%; Student's paired *t* test, *P* < 0.01). One-way ANOVA showed an effect of the treatment between groups (*F*= 6.803, *P* < 0.01); Holm–Sidak *post hoc* analysis showed a significant difference between the DEA/NO group and the DEA/NO + weak 5 Hz-LFS group (*P* < 0.05) and between the weak 5 Hz-LFS group and the DEA/NO + weak 5 Hz-LFS group (*P* < 0.05), but not between the DEA/NO group and the weak 5 Hz-LFS group (*P* > 0.05). To verify that DEA/NO + weak 5 Hz-LFS LTD was sGC dependent, the same protocol was applied in presence of the selective sGC antagonist NS2028 (1 μm), resulting in the blockade of LTD (Fig. 2*H*; *n*= 9, 104.0 ± 7.9%, *P* > 0.05). No significant difference was observed between groups treated with DEA/NO + weak 5 Hz-LFS LTD in the presence or absence of NS2028 (1 μm; Student's unpaired *t* test, *P* > 0.05). None of the drugs applied affected basal synaptic transmission. These results further indicate the potential importance of NO/sGC-dependent transmission in induction of LTD in the rat Prh.

### No role for NO signalling in LTP in perirhinal cortex

**Figure 3:**
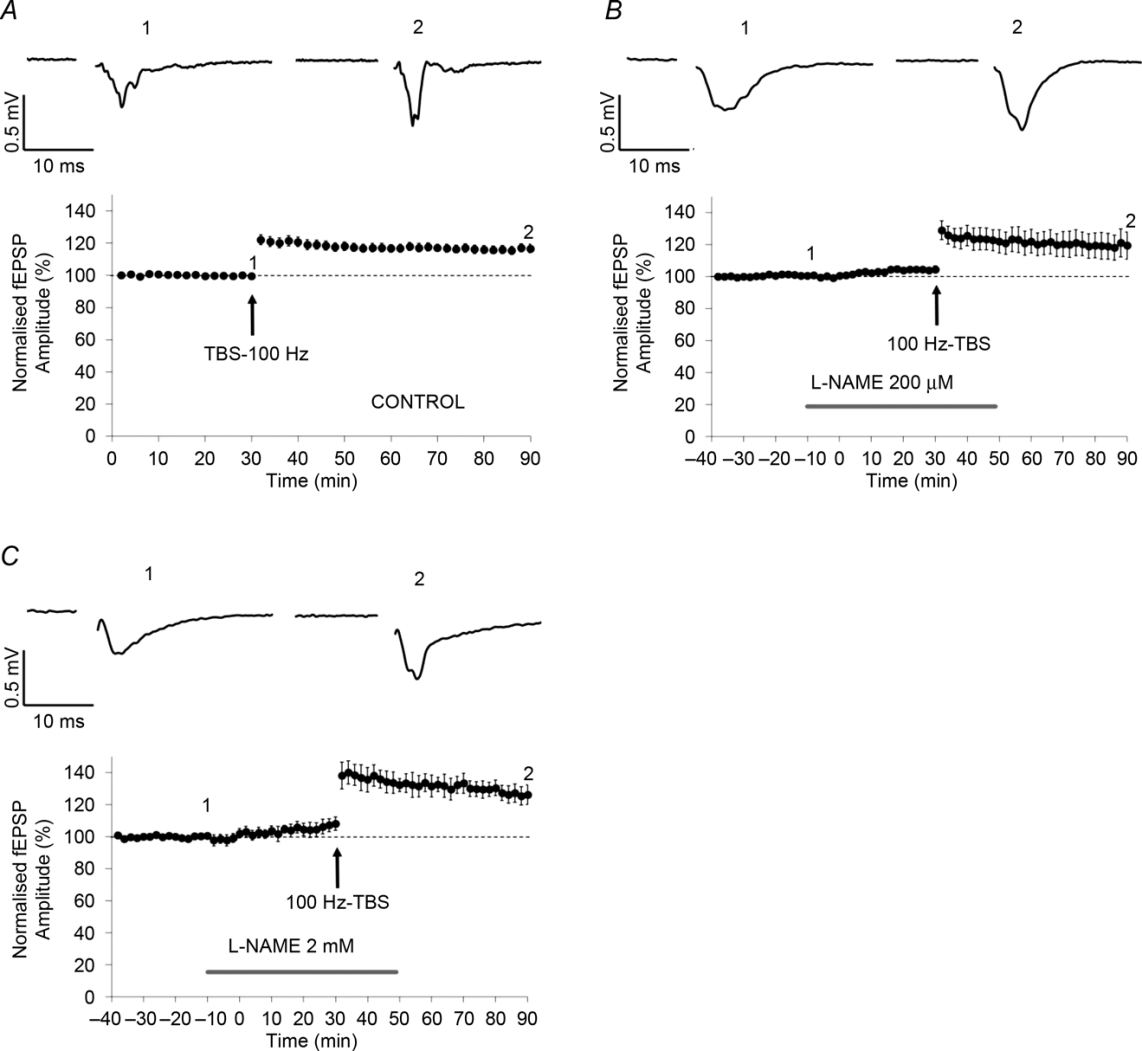
Nitric oxide synthase is not involved in 100 Hz theta-burst stimulation (TBS)-induced long-term potentiation (LTP) The application of 100 Hz-TBS resulted in the induction of a robust and prolonged LTP (*A*; *n*= 30, Student's paired *t* test, *P* < 0.01). The pre-application of the non-selective NOS inhibitor l-NAME did not affect the induction of LTP at both lower (200 μm, *B*; *n*= 5, Student's paired *t* test, *P* < 0.01) and higher concentrations (2 mm, *C*; *n*= 5, Student's paired *t* test, *P* < 0.01).

The application of 100 Hz theta-burst stimulation (100 Hz-TBS) has previously been reported to result in the induction of sustained and stable LTP in both adult and juvenile rats (Bilkey, 1996; Aicardi *et al.* 2004). Consistent with these observations, in this study we observed that 100 Hz-TBS resulted in the induction of LTP (100 Hz-TBS-LTP; Fig. 3*A*; *n*= 30, 116.6 ± 2.7%, Student's paired *t* test, *P* < 0.01). To investigate the potential role of NO-dependent signalling in LTP induction, the NOS antagonist l-NAME was pre-applied. The application of l-NAME did not affect the induction of LTP at either 200 μm (Fig. 3*B*; *n*= 5, 60 min follow-up, 119.5 ± 8.6%, Student's paired *t* test, *P* < 0.01) or 2 mm (Fig. 3*C*; *n*= 5, 126.3 ± 6.0%, Student's paired *t* test, *P* < 0.01). No significant difference was observed between each treatment and controls (one-way ANOVA, *F*= 2.461, *P* > 0.05). None of the drugs applied affected basal synaptic transmission. These results suggest that NO-dependent transmission is not required for induction of LTP in rat Prh.

### Endocannabinoid neurotransmission and the induction of LTD and LTP in perirhinal cortex

In contrast to the lack of effect of NOS inhibition on LTP, we found that pre-application (20 min before 100 Hz-TBS) of the CB1 selective antagonist AM251 (1 μm) resulted in the complete blockade of LTP (Fig. 4*A*; *n*= 8, 94.4 ± 2.8%; Student's paired *t* test, *P* > 0.05) compared with vehicle (0.01% EtOH) controls (Student's unpaired *t* test, *P* < 0.001). Recent studies have suggested that anandamide, the endogenous CB agonist, may produce plasticity through actions on TRPV1 receptors (Chávez *et al.* 2010; Grueter *et al.* 2010). Therefore, we performed experiments in the presence of the TRPV1 antagonist capsazepine. In these experiments, the fEPSP in 10 μm capsazepine-treated slices over the first 30 min after the 100 Hz-TBS application was smaller than in vehicle (0.01% DMSO)-treated control slices. However, there was no effect on the magnitude of LTP at later time points (Fig. 4*B*; *n*= 6, two-way ANOVA Veh *vs.* capsazepine, *F*= 14.220, *P* < 0.001). Holm–Sidak *post hoc* analysis showed the following interactions between treatments at the following considered time points: 30 min, *P*= 0.664; 32 min, *P*= 0.016; 60 min, *P*= 0.007; and 90 min, *P*= 0.092.

The role of CB1 signalling in the induction of CCh-LTD and 5 Hz-LTD was also evaluated. Pre-application of the CB1 selective antagonist AM251 (1 μm) did not block CCh-LTD (Fig. 4*C*; *n*= 7, 82.3 ± 4.7%, one-way repeated measures ANOVA, *P* < 0.01) compared with vehicle controls (0.1% EtOH, *n*= 5, 85.5 ± 2.9%, Student's unpaired *t* test, *P* > 0.05). Furthermore, no effect of CB1 inhibition on the acute phase of CCh application was observed (tested at the last time point of CCh application; see Table 1 for values). Likewise, pre-application of the CB1 selective antagonist AM251 (1 μm) did not affect the induction of 5 Hz-LTD (Fig. 4*D*; *n*= 5, 78.9 ± 6.5%, Student's paired *t* test, *P* < 0.01) compared with vehicle-treated controls (0.1% EtOH, *n*= 6, 84.2 ± 1.3%, Student's unpaired *t* test, *P* > 0.05). Neither AM251 nor capsazepine affected basal synaptic transmission.

**Figure 5:**
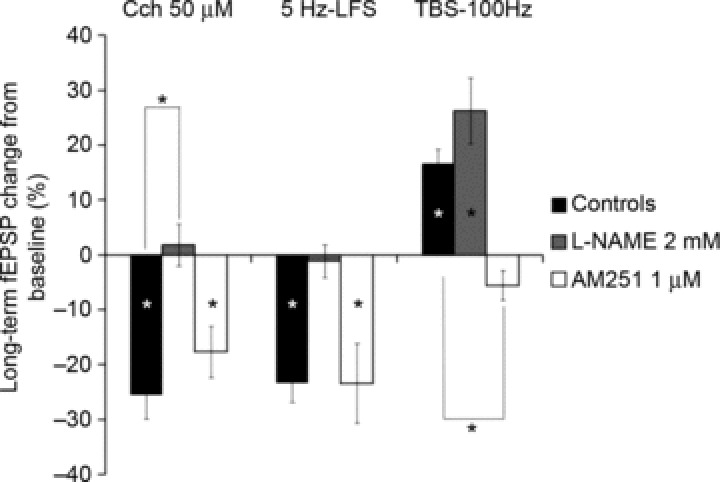
Exclusive and respective involvement of NO and endocannabinoids in Prh LTD and LTP This figure summarizes the role of NO and endocannabinoid signalling in Prh long-term synaptic plasticity. Both CCh-LTD and 5 Hz LFS-LTD are blocked by l-NAME, a NOS blocker, but not affected by AM251, a CB1 antagonist. Conversely, 100-Hz TBS-LTP is blocked by AM251, but not by l-NAME. *P < 0.05.

Taken together, these results suggest that eCB-mediated signalling may be important for LTP in Prh, reinforcing the recent idea of CB1 involvement in potentiation-like phenomena, as suggested by some recent studies (Abush & Akirav, 2010; Navarrete & Araque, 2010). In addition, these data suggest that TRPV1 may play some role in short-term but not long-term potentiation in Prh. The effects of NOS inhibition and CB1 receptor antagonism are summarized in Fig. 5.

### Role of nitric oxide signalling in perirhinal cortex-dependent acquisition of visual recognition memory

**Figure 6:**
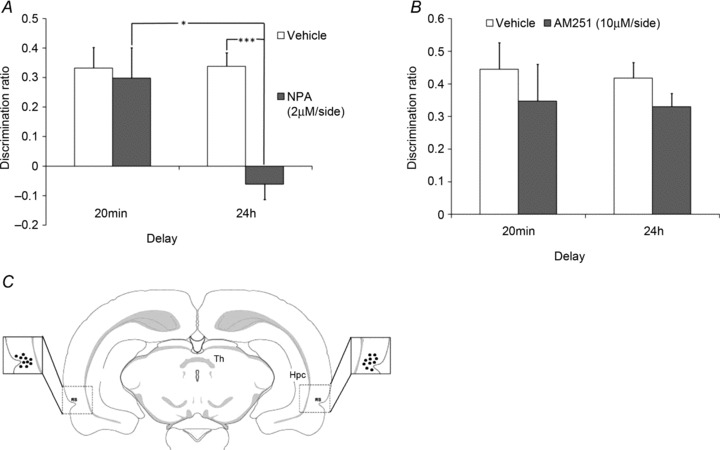
Involvement of NO but not endocannabinoids in visual recognition memory acquisition in adult rats *A*, bilateral infusion of the nNOS selective antagonist NPA (2 μm) in adult rat Prh impaired long-term (24 h) but not short-term (20 min) visual recognition memory. For control animals, the discrimination ratio was significantly different from zero (i.e. discrimination between novel and familiar) at both delays, whereas for NPA-treated animals the discrimination ratio was significantly different from zero at 20 min but not at 24 h. *P < 0.01 difference between the 20 min and 24 h delay within NPA-treated animals; ***P < 0.001, difference between vehicle- and NPA-treated animals at the 24 h delay. *B*, infusion of the CB1 selective antagonist AM251 (10 μm) in the Prh does not affect visual recognition memory at both delays. Data are presented, for each group, as means (±SEM). The discrimination ratio is the proportion of additional time spent exploring a novel rather than a familiar object. *C*, verification of placement of the cannulae. Each dot represents the location of a cannula tip (shown in the box expanded from a schematic brain section) in a different rat (*n*= 10). Abbreviations are as follows: Hpc, hippocampus; RS, rhinal sulcus; and Th, thalamus.

Bilateral infusion of the selective antagonist for nNOS, NPA (2 μm), into the Prh significantly impaired long-term but not short-term visual object recognition memory. Two-way ANOVA [within-subject factors, drug (vehicle *vs.* NPA); delay (20 min *vs.* 24 h)] revealed a significant drug-by-delay interaction [*F*(1,20) = 12.99, *P* < 0.01] and a significant effect of drug [*F*(1,20) = 18.18, *P* < 0.001] but no significant effect of delay [*F*(1,20) = 4.09, *P* > 0.05]. Analyses of the significant main effects revealed that the NPA-infused animals were significantly impaired compared with the vehicle-infused animals at the 24 h (*P* < 0.001; Fig. 6*A*) but not the 20 min delay (*P* > 0.1; Fig. 6*A*).

Additional analysis confirmed that the vehicle-infused animals discriminated between the novel and familiar objects at both delays tested [20 min *t*(9) = 4.50, *P* < 0.001; 24 h *t*(11) = 7.07, *P* < 0.001]; in contrast, the NPA-infused animals showed discrimination between the novel and familiar object only at the 20 min delay [*t*(9) = 2.76, *P* < 0.05] but not at the 24 h delay [*t*(11) =−1.13, *P* > 0.1].

### Exploration in the sample and test phases

Analysis of the time taken to complete the sample phase and the amount of exploration completed in the sample and test phases revealed no significant interaction between treatment and delay (for all *F* < 1.0, *P* > 0.1) and no significant effect of drug [time to complete sample phase, *F*(1,20) = 2.78, *P* > 0.1; exploration in sample phase, *F* < 1.0, *P* > 0.1; and exploration in test phase *F* < 1.0, *P* > 0.1]. However, there was a significant effect of delay on the amount of exploration completed in the test phase [*F*(1,20) = 4.88, *P* < 0.05], which reflected the fact that both vehicle- and NPA-infused animals spent significantly more time exploring the objects at the 20 min delay than the 24 h delay; there was no significant effect of delay on the amount of time taken to complete the sample phase (*F* < 1.0, *P* > 0.1) and the amount of exploration completed in the sample phase [*F*(1,20) = 2.36, *P* > 0.1; see Table 2 for means].

**Table 2:** Effect of the neuronal nitric oxide synthase selective antagonist NPA and CB1 selective antagonist AM251 on general exploratory behaviour

Infusion	Delay	Time to complete acquisition phase (s)	Total exploration in acquisition phase (s)	Total exploration in test phase
Vehicle	20 min	190 ± 14	34 ± 3	33 ± 3
NPA	(*n*= 10 per group)	210 ± 13	34 ± 2	31 ± 2
		*F*(1,20) < 1.0; n.s.	*F*(1,20) < 1.0; n.s.	*F*(1,20) < 1.0; n.s.
Vehicle	24 h	214 ± 11	36 ± 1	26 ± 1
NPA	(*n*= 10 per group)	227 ± 6	35 ± 1	27 ± 2
		*F*(1,20) < 1.0; n.s.	*F*(1,20) < 1.0; n.s.	*F*(1,20) < 1.0; n.s.
Vehicle	20 min	174 ± 15	40 ± 0.1	30 ± 3
AM251	(*n*= 10 per group)	191 ± 17	38 ± 1	34 ± 3
		*F*(1,18) < 1.0; n.s.	*F*(1,18) < 1.0; n.s.	*F*(1,18) < 1.0; n.s.
Vehicle	24 h	169 ± 20	36 ± 2	25 ± 3
AM251	(*n*= 10 per group)	154 ± 18	39 ± 0.7	25 ± 2
		*F*(1,18) < 1.0; n.s.	*F*(1,20) < 1.0; n.s.	*F*(1,18) < 1.0; n.s.

No significant (n.s.) differences in total exploration times were observed between control and treated animals; hence, the drugs had no significant effect on general exploratory behaviour.

### Role of endocannabinoid signalling in perirhinal cortex-dependent acquisition of visual recognition memory

Bilateral infusion of the CB1 selective antagonist AM251 (10 μm) into the Prh had no effect on short-term or long-term visual object recognition memory (Fig. 6*B*). Analysis of the discrimination ratios at test revealed a non-significant drug-by-delay interaction [*F*(1,18) < 1.0, *P* > 0.1], a non-significant effect of drug [*F*(1,18) < 1.0, *P* > 0.1] and no significant effect of delay [*F*(1,18) < 1.0, *P* > 0.1].

Additional analysis confirmed that both the vehicle- and the AM251-infused animals showed significant discrimination between the novel and familiar objects at both tested delays [20 min AM251, *t*(9) = 2.93, *P* < 0.05; 20 min Veh, *t*(9) = 5.19, *P* < 0.001; 24 h AM251 *t*(9) = 7.66, *P* < 0.001; and 24 h Veh, *t*(9) = 8.28, *P* < 0.001]. Absolute exploration time values of the novel and familiar objects are reported in Table 3.

**Table 3:** Absolute exploration times for the novel and familiar object after 20 min or 24 h delay in the presence of NPA, AM251 or respective vehicles

Infusion	Delay	Novel object exploration (s)	Familiar object exploration (s)
Vehicle	20 min	22.1 ± 1.84	11.4 ± 1.54
NPA	(*n*= 10 per group)	20.0 ± 2.21	11.1 ± 1.95
Vehicle	24 h	17.8 ± 1.29	8.6 ± 0.64
NPA	(*n*= 10 per group)	13.0 ± 1.12	14.4 ± 0.94
Vehicle	20 min	21.3 ± 1.82	8.8 ± 2.14
AM251	(*n*= 10 per group)	23.1 ± 2.80	10.5 ± 1.52
Vehicle	24 h	18.0 ± 2.43	7.1 ± 1.09
AM251	(*n*= 10 per group)	16.7 ± 1.32	8.4 ± 0.73

### Exploration in the sample and test phases

Analysis of the time taken to complete the sample phase and the amount of exploration completed in the sample and test phases revealed no significant interaction between treatment and delay [time to complete sample phase, *F*(1,18) < 1.0, *P* > 0.1; exploration in sample phase, *F*(1,18) = 4.36, *P* > 0.05; and exploration in test phase, *F*(1,18) < 1.0, *P* > 0.1] and no significant effect of drug [for all *F*(1,18) < 1.0, *P* > 0.1]. Also, there was no significant effect of delay on the time taken to complete the sample phase and the amount of exploration completed in the sample [time to complete sample phase, *F*(1,18) = 2.16, *P* > 0.1; and exploration in sample phase, *F*(1,18) < 1.0, *P* > 0.1]; however, there was a significant effect of delay on the amount of exploration completed in the test phase [*F*(1,18) = 7.42, *P* < 0.05], which reflected the fact that both vehicle- and AM251-infused animals spent significantly more time exploring the objects at the 20 min delay compared with the 24 h delay (see Table 2 for means).

### Histological verification of cannula positions

Cannula locations were checked against standardized sections of the rat brain (see Methods). All animals had the tips of their cannulae within the Prh from bregma −5.5 to −4.5 mm (Paxinos & Watson, 1986; Shi & Cassell, 1999; Fig. 6*C*)

## Discussion

The results of this study demonstrate dissociation between retrograde signalling mechanisms in LTD and LTP in Prh. Thus, LTP relies on cannabinoid but not NO signalling, whilst LTD relies on NO but not eCB signalling. Critically, the results also establish, for the first time, that NO, but not eCB, signalling is important in object recognition memory acquisition.

Evidence from a number of studies in different brain regions supports a role for NO as a retrograde messenger in synaptic plasticity, for example: in LTD at the parallel fibre to Purkinje cell synapse (Shin & Linden, 2005); LTD in prefrontal cortex (Huang & Hsu, 2010); hippocampal LTD and LTP (Arancio *et al.* 1995; Reyes-Harde *et al.* 1999; Bon & Garthwaite, 2003; Zhang *et al.* 2006); and visual cortex LTP (Haghikia *et al.* 2007). In addition, the nNOS has been shown to be expressed ubiquitously in Prh and it is particularly dense in layer II/III (Liu *et al.* 2003*b*; Lein *et al.* 2007). Our results are the first to demonstrate that LTD in Prh relies on NO. These results were obtained with two different NOS inhibitors, l-NAME and NPA, suggesting that the block of LTD is not due to non-specific pharmacological effects of the inhibitors. It has been reported that NPA is a selective neuronal NOS inhibitor (Zhang *et al.* 1997) and has little effect on endothelial NOS (eNOS). However, the selectivity of NPA has been challenged (Pigott *et al.* 2012) and therefore it is still not possible to conclude definitively that the effects on LTD are most likely to be due to synaptic production of NO rather than to effects of NO derived from blood vessels. Our results also demonstrate a lack of effect of NOS inhibitors on LTP in Prh. This result is important for two reasons; firstly, it further indicates that block of LTD by NOS inhibition is unlikely to be due to non-specific general effects on synaptic function and plasticity; and secondly, this result suggests that NO is not a ubiquitous retrograde messenger for all forms of synaptic plasticity in Prh.

The reasons why NO might be important in LTD but not in LTP are not clear, but might reflect the different transmitter and receptor mechanisms that are involved in the induction of LTD and LTP. In Prh, metabotropic glutamate receptors, muscarinic receptors and voltage-gated calcium channels (VGCCs) are involved in the induction of LTD, but not in the induction of LTP (Jo *et al.* 2006, 2008; Massey *et al.* 2008; Seoane *et al.* 2009). Thus, it is possible that NOS is preferentially activated by these transmitters and/or calcium influx via VGCCs, leading to a specific role of NO in LTD.

CB1 receptors are expressed ubiquitously in Prh, particularly in layer II/III (Tsou *et al.* 1998; Liu *et al.* 2003*a*; Lein *et al.* 2007), but little is known about their function in this cortical region. The role of eCBs as retrograde messengers that depress transmitter release in suppression of inhibition or suppression of excitation is now well established (Alger 2002; Kano *et al.* 2008). In addition, there is much evidence that eCB signalling is also important in synaptic plasticity, especially in LTD mechanisms (reviewed by Heifets & Castillo, 2009). In contrast, however, evidence for a role of CB1 receptors in LTP is limited. In this context, therefore, it was somewhat surprising to find that CB1 inhibition prevented the induction of perirhinal LTP but did not affect CCh-LTD or activity-dependent LTD in Prh. Clearly, the block of LTP in our study indicates that the lack of effect of CB1 inhibition on LTD was not due to ineffectiveness of the CB1 inhibitor or lack of CB1 receptors or associated signalling machinery in the Prh. Recently, it has been shown that intraperitoneal injection of AM251 in rats impaired LTP induction at the Schaffer collateral to CA1 synapses, while an inhibitor of reuptake and breakdown of the eCBs facilitated LTP (Abush & Akirav, 2010). These results suggest that a role for CB1 receptors in LTP in other brain regions may have been overlooked and needs further scrutiny.

The precise mechanisms by which eCBs may produce LTP in Prh are not clear. One possible explanation is that presynaptic CB1 receptors depress GABA release during high-frequency stimulation (Alger, 2002; Kano *et al.* 2008) and this depression of inhibition facilitates LTP induction. Other possible explanations also exist for the effects of CB1 inhibitors on LTP. A recent study has shown that the activation of CB1 receptors on astrocytes can stimulate the release of glutamate that acts on presynaptic metabotropic glutamate receptors, resulting in LTP (Navarrete & Araque, 2010); whether a similar mechanism exists in Prh is not known. Recent studies suggest that eCBs may act through TRPV1 receptors in the induction of synaptic plasticity (Chávez *et al.* 2010; Grueter *et al.* 2010). Given that the CB1 inhibitor AM251 blocked LTP, we investigated the effect of the TRPV1 inhibitor capsazepine and found an effect on short-term potentiation but not on LTP. These results suggest that the involvement of eCBs in 100 Hz-TBS-induced synaptic potentiation may be through a combination of TRPV1 receptor and CB1 receptor activation. The precise mechanisms by which TRPV1 receptors contribute to short-term potentiation will require much further investigation and are outside the scope of the present study.

In the behavioural experiments reported in this study, we show that infusion of NPA, a selective NOS inhibitor, directly into Prh blocked the acquisition of long-term, but not short-term, object recognition memory. The memory impairments we report are not likely to be due to generalized effects of the NOS inhibitor, because no differences were observed in the total exploration times in each phase of the task for both drug-treated and vehicle-treated animals.

The impairment of long-term, but not short-term, familiarity discrimination by NOS inhibition is similar to the pattern of impairment found previously following the antagonism of NMDA receptors (Barker *et al.* 2006*b*), metabotropic glutamate receptors (Barker *et al.* 2006*a*) or VGCCs (Seoane *et al.* 2009) in the Prh. Thus, it is possible that the nNOS signalling important in recognition memory is triggered by activation of such glutamate receptors and/or VGCCs.

Previous work has also suggested that there may be a role for NO signalling in recognition memory. The systemic administration of the non-selective NOS inhibitor l-NAME after the training phase resulted in impairment of visual recognition memory when tested at 24 but not at 1 h (Boultadakis *et al.* 2010), while the systemic administration of the phosphodiesterase inhibitor sildenafil resulted in increased retention of recognition memory in rats (Prickaerts *et al.* 2002) and mice (Rutten *et al.* 2006). However, the systemic administration of drugs in these studies does not allow one to ascribe any specific role to NO in Prh.

In the CNS, NO can be produced by the following three NOS isoforms: eNOS, constitutively expressed in the endothelium; nNOS, constitutively expressed in neurones and glia; and inducible NOS (iNOS), mainly expressed in glial cells exclusively in response to pathogenic stimuli. Typically, it is thought that nNOS and eNOS are involved in physiological NO-mediated functions (Garthwaite, 2008; reviewed by Steinert *et al.* 2010). Therefore, in physiological conditions it is important to differentiate between endothelial and neuronal NOS production. However, given the debate over the selectivity of NPA for nNOS *vs.* eNOS (see Zhang *et al.* 1997; Pigott *et al.* 2013), it is still not possible to draw strong conclusions about whether synaptically produced NO or endothelium-derived NO is more important in the encoding of familiarity discrimination.

Various lines of evidence have previously suggested that CB1 receptors are important in learning and memory (Marsicano *et al.* 2002; Varvel *et al.* 2007). Thus, exogenous activation of CB1 receptors has been shown to impair hippocampal and prefrontal cortex learning, whilst learning and memory are enhanced by CB1 antagonists or in CB1 knockout mice (Riedel & Davies 2005; Egerton *et al.* 2006; Lutz, 2007). More specifically, CB1 knockout mice had improved memory performance in a 24 h delay object recognition task (Reibaud *et al.* 1999; Lutz, 2007). In contrast, however, we did not identify a role for CB1 receptor signalling in Prh-dependent learning in the present experiments, and several issues may explain these differences. Firstly, the results in the study by Reibaud *et al.* (1999) were based on a global CB1 knockout; therefore, the behavioural effects observed may be due to effects outside of the Prh. Secondly, there are procedural differences in the assessment of recognition memory between the two studies. In the study by Reibaud *et al.* (1999), only one object was presented in the sample phase and two objects were presented in the test phase. Thus, a spatial memory component that does not involve Prh may have been introduced into the design of that experiment.

Importantly, the dissociation between the roles of NO- and eCB-dependent signalling in synaptic plasticity allows us to speculate about the roles of LTP and LTD induction in familiarity discrimination. Using these tools, we are able selectively to block one specific mechanism underlying LTP in Prh *in vivo* and find that this has no effect on familiarity discrimination. In contrast, the block of an LTD-related mechanism prevented familiarity discrimination, in line with previous work (Griffiths *et al.* 2008; Seoane *et al.* 2009).

In conclusion, the results of this study provide the first demonstration of the specific and respective role of NO and eCBs in perirhinal LTD and LTP. Critically, we also demonstrate that NO, but not eCB signalling, plays a key role in Prh-dependent visual recognition memory.

## Abbreviations

aCSF: artificial cerebrospinal fluid
AM251: 1-(2,4-dichlorophenyl)-5-(4-iodophenyl)-4-methyl-*N*-(1- piperidyl)pyrazole-3-carboxamide
CB1: cannabinoid receptor 1
CCh: carbachol
eNOS: endothelial nitric oxide synthase
DEA/NO: diethylamine-NONOate
eCBs: endocannabinoids
fEPSP: field excitatory postsynaptic potential
iNOS: inducible nitric oxide synthase
LFS: low-frequency stimulation
l-NAME: l-*N*^G^-nitroarginine methyl ester hydrochloride
LTD: long-term depression
LTP: long-term potentiation
nNOS: neuronal nitric oxide synthase
NOS: nitric oxide synthase
NPA: *N*^G^-propyl- l-arginine
NS2028: 4*H*-8-bromo-1,2,4-oxadiazolo[3,4-d]benz[b][1,4]oxazin-1-one
Prh: perirhinal cortex
sGC: soluble guanylate cyclase
TBS: theta-burst stimulation
TrpV1: transient receptor potential cation channel subfamily V member 1
VGCC: voltage-gated calcium channel
